# γδT cells regulate chronic airway inflammation and development of airway remodelling

**DOI:** 10.1111/cea.12395

**Published:** 2014-10-21

**Authors:** J R Murdoch, L G Gregory, C M Lloyd

**Affiliations:** Leukocyte Biology Section, Faculty of Medicine, National Heart and Lung Institute, Imperial College LondonLondon, UK

**Keywords:** airway remodelling, allergic inflammation, Th2 cells, γδT cells

## Abstract

**Background:**

γδT cells play a crucial immunoregulatory role in the lung, maintaining normal airway tone and preventing hyperresponsiveness to innocuous allergen. During acute inflammatory episodes, γδT cells promote resolution of acute inflammation. However, their contribution to inflammation-associated airway remodelling remains unexplored. Here we investigate the effects of γδT cell blockade on established allergic airway inflammation and development of remodelling.

**Methods:**

Sensitised mice were exposed to prolonged ovalbumin challenge or continuous house-dust mite exposure to induce chronic inflammation and remodelling. Functional blocking anti-TCRδ antibody was administered therapeutically, and parameters of airway inflammation and remodelling were examined.

**Results:**

Therapeutic blockade of γδT cells prevented the typical resolution of acute airway inflammation characterised by elevated eosinophil and Th2 cell numbers. Moreover, the lung displayed exacerbated airway remodelling, typified by excess peribronchiolar collagen deposition.

**Conclusions:**

These results demonstrate a unique role for γδT cells in constraining allergen-induced airway remodelling. Manipulating the γδT cell compartment may therefore contribute to strategies to prevent and treat remodelling.

## Introduction

In recent decades, allergic asthma has emerged as a major global health burden. It is a chronic inflammatory disease of the airways that results from environmental interactions, complex immunobiology and genetic factors. Airway hyperreactivity (AHR), inflammation and self-amplifying mediator release define the principal pathological features of the disease 1–3. Allergen challenge leads to an influx of large quantities of inflammatory eosinophils to the conducting airways. Products of these cells (enzymes, profibrotic mediators including transforming growth factor-β) contribute to collateral lung tissue damage 4,5. Persistent and unresolving airway inflammation cumulates in a variety of alterations to airway architecture. Indeed, extensive injury to the airway epithelium and activation of epithelial cells and underlying fibroblasts are commonly described in patients with asthma 1,3,6. The resulting pathophysiological changes to airway architecture, referred to as ‘remodelling’, encompass confounding subepithelial fibrosis through deposition of collagen and dysregulated matrix turnover 7. This poorly understood process leads to a progressive decline in lung function which correlates with disease severity and poor response to therapy 8. Appropriate regulation of inflammatory infiltrates represents a key strategy to limit allergic immunopathology associated with the effector lymphocyte responses.

γδT cells constitute the major T cell component of lung epithelium in the steady state 9,10. Although γδT cells exhibit some characteristics of adaptive immunity, they lack the antigen specificity of classical αβT cells 11, instead their functions are predominantly stress surveillance in response to signals from their surrounding microenvironment and such stress signals are a substantial driver of immune responses 12. In contrast, regulatory functions of γδT cells have been demonstrated during established disease to promote wound healing 13,14. Interestingly, γδT cells are elevated in the airways of asthmatics 15, and a regulatory γδT cells population elicited in response to allergen exposure has been observed in both mice and rats 16,17. We recently demonstrated the vital contribution of IL-17^+^γδT cells to resolution of acute allergic airway inflammation 18. However, the impact of these cells on the development of remodelling has not yet been investigated. We hypothesised that by providing a requisite inflammatory resolution mechanism in the airway, γδT cells indirectly confound the remodelling processes that are caused, in part, through repeated cycles of damage and repair. We have used models of prolonged allergen challenge to induce chronic inflammation-driven remodelling and evaluated the role of γδT cells in this process. Asthmatic patients present with established disease; therefore, understanding regulatory factors influencing the course and outcome of chronic disease is important. In contrast to previous studies employing prophylactic measures to investigate γδT cells, a therapeutic approach was undertaken following induction of established allergic airway disease. We determined that depletion of γδT cells exacerbated remodelling as a consequence of impaired inflammatory resolution.

## Methods

### Animals

Female BALB/c mice, purchased from Harlan Olac Ltd (Bicester, UK), were housed at Imperial College animal facility with food and water supplied *ad libitum*. UK Home Office guidelines for animal welfare based on the Animals (Scientific Procedures) act 1986 were observed.

### *In vivo* experimental protocol

*Sensitisation and airway challenge* BALB/c mice were sensitised intraperitoneally (i.p.) using 0.01 mg/mouse OVA (Sigma, Poole, UK) in 0.2 mL Alum (Alu-Gal-Ser, Serva Electrophoresis) on d0 and d12. Control mice were sham sensitised using an equivalent volume of PBS/Alum. Acute (day 24) and chronic (day 35 and 55) airway disease was induced in using an established protocol of extended airway challenge 3. Mice also received 25 μg HDM extract (*Dermatophagoides pteronyssinus* in PBS) (Greer laboratories, Lenoir, North Carolina, USA) or 25 μL PBS intranasally 5 days a week for 5 weeks. Disease parameters were assessed in animals sacrificed by exsanguination under terminal anaesthesia (100 mg/kg ketamine (Fort Dodge Animal Health) and 10 mg/kg domitor (Pfizer) 24 h after final allergen challenge.

*Administration of function blocking anti-TCR*δ *mAb* 100 μL anti-TCR-δ (200 μg/mL), hamster monoclonal antibody against the γδTCR (a gift from L. Lefrancois) was injected into the tail vein twice weekly from either day 24 (protocol A) or 40 (protocol B) onwards in the OVA model and from week 3 onwards in the HDM model. Thorough blockade was ensured at each endpoint by flow cytometric analysis with anti-γδT cell antibody from a different clone. Sham treatment was accomplished with an equivalent volume of hamster Ig (Jackson Laboratories).

### Measurement of AHR

AHR was determined by direct measurements of resistance and compliance in anesthetised and tracheostomised mice in response to inhaled methacholine (MCh; Sigma, Cambridge, UK) at concentrations of 3–100 mg/mL for 1 min in an EMMS system (EMMS, Hampshire, UK) in a modified version of previously described methods 19,20.

### Cell recovery

*Airway Lumen* Bronchoalveolar lavage (BAL) was performed by three flushings of the lung with 0.4 mL PBS via a tracheal cannula resulting in the recovery of 1 mL BAL fluid. *Lung parenchyma* one lobe of lung tissue was digested in complete media (RPMI + 10% FCS, 2 mm L-Glutamine, 100 U/mL Penicillin/Streptomycin) containing 0.15 mg/mL collagenase (Type D, Roche Diagnostics) and 25 μg/mL DNase (Type 1, Roche Diagnostics). Cells were recovered by filtration through a 70-μm nylon sieve (Falcon) and resuspended in 1-mL complete media.

### Quantification of eosinophils

Cells from the BALF and lung were counted and pelleted onto glass slides by cytocentrifugation (5 × 10^4^ cells/slide). Differential cell counts were performed on Wright-Giemsa-stained (Thermo) cytospins. Percentages of eosinophils, lymphocyte/mononuclear cells, neutrophils and macrophages were determined by counting their number (∼400 total cells counted per slide) and dividing this number by the total number of cells counted. To obtain absolute numbers of eosinophils, the percentage was multiplied by the total number of cells obtained in the lavage fluid and lung homogenate.

### Lung tissue histopathology

Four-μm paraffin-embedded lung sections were stained with haematoxylin and eosin for evaluation of eosinophilic infiltrates.

### Assessment airway remodelling

Peribronchiolar collagen deposition was quantified on Sirius Red-stained sections viewed under polarised light using Scion-Image software (Scion Corporation) 21. The mean density of collagen staining was calculated (pixels/μm^2^). Digital photographs representative of bronchioles from each group were taken. Paraffin sections were stained with rabbit anti-mouse proliferating cell nuclear antigen (PCNA) (Abcam, Cambridge, UK) and α-smooth muscle actin (α-SMA) (Abcam) using an avidin/biotin staining. Epithelial cell proliferation was expressed as the % PCNA^+^ cells among total bronchiolar epithelial cells counted. The thickness of the α**-**SMA^+^ peribronchiolar smooth muscle layer was calculated by multiple measurements perpendicular to the basement membrane.

### Total lung collagen assay

Total collagen was assessed in lung homogenate using a Sircoll dye kit (Biocolor Ltd) according to the manufacturer's protocol.

### Flow cytometric analysis

BAL and lung tissue leukocyte suspensions were stained with anti-mouse CD3, anti-mouse CD4, anti-mouse-TCRδ, anti-mouse Vγ4, anti-mouse IL-17A (BD Pharmingen, Oxford, UK), anti-mouse T1/ST2 (Morwell Diagnostics, Zurich, Switzerland), α-GalCer (Axxora Biochemicals, Farmingdale, NY, USA) loaded CD1d tetramers (ProImmune Ltd., Oxford, UK) or relevant isotype controls. Flow cytometric analysis was performed using a FACSCalibar™ (Becton Dickenson, Oxford, UK) using CellQuest software.

### Lung mediator analysis

One lobe of the lung was homogenised at 50 mg/mL in Hanks buffered salt solution (HBSS) containing protease inhibitor cocktail. Samples were centrifuged at 570 g and cytokines measured in the supernatant (S1) fraction. Cytokines are reported in mg/mL of the S1 fraction. Paired antibodies for murine IL-4, IL-5 (BD Pharmingen) and eotaxin-1/CCL11 (R&D Systems) were used in standardised sandwich ELISAs according to the manufacturer's protocol. Lavage IL-13 was measured using a kit according to manufacturer's instructions (R&D Systems).

### SEAP TGF-β Bioassay 22

F11 cells (A gift from T. Wyss-Coray) seeded at 4.10 × 10^4^ cells/well in 96-well flat-bottom tissue culture plates were incubated with 50 μL activated homogenised lung sample (activated by addition of 2.5 μL 6 m HCl for 10 min followed by neutralisation to pH 7.4 with 6 m NaOH) for 24 h. Relative luminescence for SEAP activity was measured using Great EscAPe SEAP Reporter system 3 (Clontech, WI, USA) according to the manufacturer's instructions.

### Data analysis

Data are expressed as mean ± SEM unless otherwise stated. Statistical significance between groups was tested using a Mann–Whitney *U*-test. *P* of < 0.05 was considered significant. Graph generation and statistical analysis were performed using prism software (version 4.00; GraphPad).

## Results

### Prolonged airway challenge elicits increased pulmonary γδT cells

**Figure 1:**
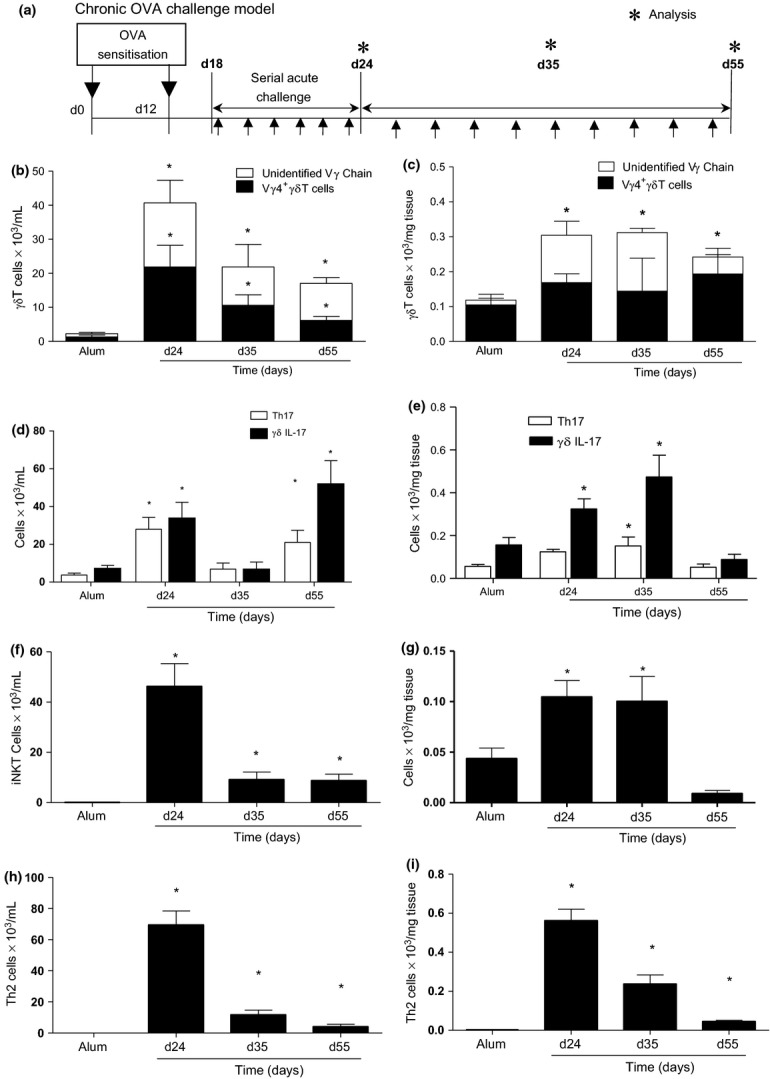
Pulmonary γδT cells are elevated following allergen exposure. Schematic OVA challenge model (a). Animals were systemically sensitised with alum/OVA (alum/PBS) followed by acute OVA challenge (days 18–24). To induce chronic inflammation and remodelling, OVA challenges were continued 3× weekly. Mice were sacrificed 24 h after final aerosol challenge on days 24, 35 and 55. Total and Vγ4^+^ γδT cells in the BAL (b) and lung (c). Th17 and IL-17^+^ γδT cells in the BAL (d) and lung (e). iNKT cells in the BAL (f) and lung (g). Th2 cells in the BAL (h) and lung (i). Data represent mean ± SEM (*n *=* *6–12). *P <* *0.05 compared to alum controls, (Mann–Whitney *U*-test). Alum controls from days 24, 35 and 55 were not significantly different between time points and were pooled for clarity.

Allergen provocation of OVA-sensitised mice elicits airway inflammation while prolonged aerosol challenge with OVA is associated with a switch to a chronic inflammatory remodelled phenotype 3. To investigate the role of γδT cells to the chronic remodelled phenotype *in vivo*, we assessed the effect of prolonged OVA challenge (depicted schematically, Fig.1a), on pulmonary leukocytes within the airway lumen and tissue compartments of OVA-sensitised mice by flow cytometry (Fig.1b). Both total and Vγ4^+^ γδT cells (which have been shown to be regulatory in the asthmatic lung 23) were significantly elevated following acute (day 24) and extended challenge (day 35 and 55) in both the bronchiolar lavage (BAL) (Fig.1b) and lung tissue (Fig.1c). Local changes in pulmonary γδT cell numbers were not reflected in lung-draining lymph nodes, peripheral lymph nodes or spleen (data not shown). There was an increase in both IL-17^+^ T cells (Th17) and IL-17^+^ γδT cells in response to OVA challenge (Fig.1d,e). The innate lymphocytes iNKT cells also increase following OVA exposure (Fig.1f,g). Th2 cells were also recruited to the lung as part of the adaptive immune response to allergen challenge (Fig.1h,i). Peak recruitment of Th2 cells (Fig.1i) and eosinophils 3 to the lung occurs on d24 and decreases over time. Interestingly, the kinetics of γδT cells during the course of allergen challenge did not reflect this and remain elevated.

### γδT cells regulate airway remodelling

**Figure 2:**
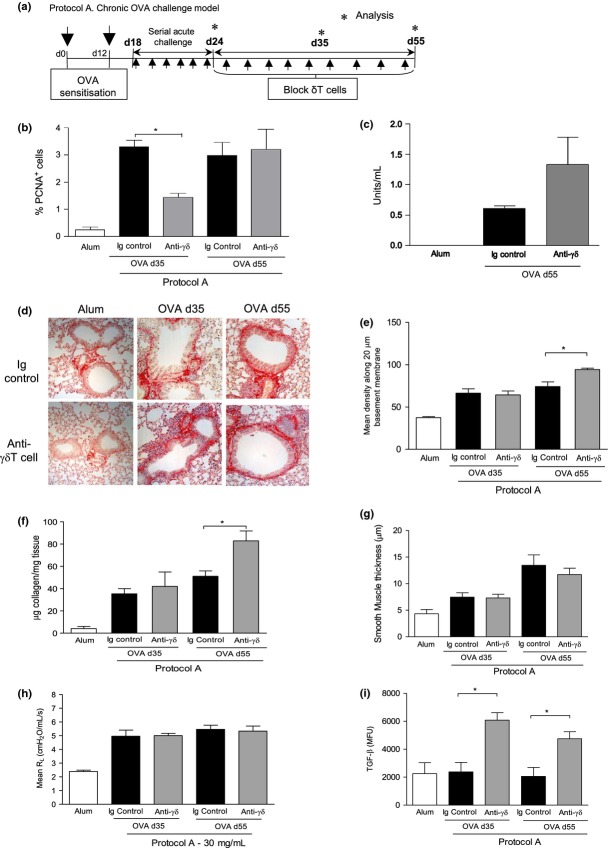
Blocking γδT cells during established inflammation promotes collagen deposition and TGF-β-mediated tissue remodelling in the allergic lung. *Protocol A* – Following sensitisation (OVA/alum i.p.) and acute aerosolised OVA challenge (day 24), female BALB/c mice were administered 100 μL of 200 μg/mL anti-TCRδ blocking mAb (or hamster Ig control) i.v. (twice weekly) and disease parameters assessed on days 35 and 55, 24 h after allergen challenge (a). % PCNA+ve epithelial cells (b). Mucous in the lung was measured by ELISA (c). Sirius Red-stained lung sections demonstrating peribronchiolar collagen deposition from Alum and OVA-treated mice treated with either anti-γδTCR or the Ig control antibody (d), original magnification 40×. Quantitative analysis of subepithelial peribronchiolar collagen density determined from Sirius Red-stained collagen in lung sections (e). Biochemical Sircol Assay of total lung collagen content (f). Peribronchiolar smooth muscle thickness (g). Airway resistance in response to 30 mg/mL methacholine challenge (h). Detection of biologically TGF-β activity was measured in lung tissue homogenate using a SEAP reporter bioassay (i). Data are expressed as mean ± SEM, *n *=* *8–12 per group. *P <* *0.05 in comparison with OVA Ig controls (Mann–Whitney *U*-test). Alum controls from days 35 and 55 were not significantly different between time points and are pooled for clarity.

To determine the contribution of γδT cells to airway remodelling, anti-TCRδ functional blocking antibody GL3 was administered therapeutically after establishment of allergic inflammation (Protocol A, Fig.2a). PCNA is an antigen expressed in the nuclei of proliferating cells in S-phase of the cell cycle. OVA challenge increased bronchiolar epithelial cell turnover/proliferation as evidenced by increased numbers of PCNA^+^ epithelial cells. Blockade of γδT cells delayed the epithelial repair response induced in response to allergen challenge (Fig.2b). OVA induces phenotypic changes of airway epithelial cells to a mucous secreting phenotype 3; however, blockade of γδT cells did not affect mucous secretion (Fig.2c). Increased deposition of collagen around the airways was evident in Sirius Red-stained lung sections of OVA-exposed mice (Fig.2d). Quantification of peribronchiolar collagen revealed anti-TCRδ antibody-treated mice had significantly more collagen fibrils around the airways by day 55 (Fig.2e). This was also confirmed by analysis of total lung collagen (Fig.2f). Although excessive extracellular matrix deposition was observed in mice treated with anti-γδTCR antibody the lack of functional γδT cells did not influence peribronchiolar airway smooth muscle mass (Fig.2g). Blocking γδT cells also did not affect airway hyperreactivity (Fig.2h) which is a function of airway smooth muscle. TGF-β is profibrotic mediator vital to development of airway remodelling in this model 3. Substantially more functionally active lung TGF-β was detected on both days 35 and 55 in treated mice using a highly sensitive bioassay (Fig.2i) 22. No differences in levels of immunoregulatory IL-10 in the lung were observed between groups (data not shown).

### γδT cells regulate resolution of airway inflammation in chronic eosinophilic airway inflammation

**Figure 3:**
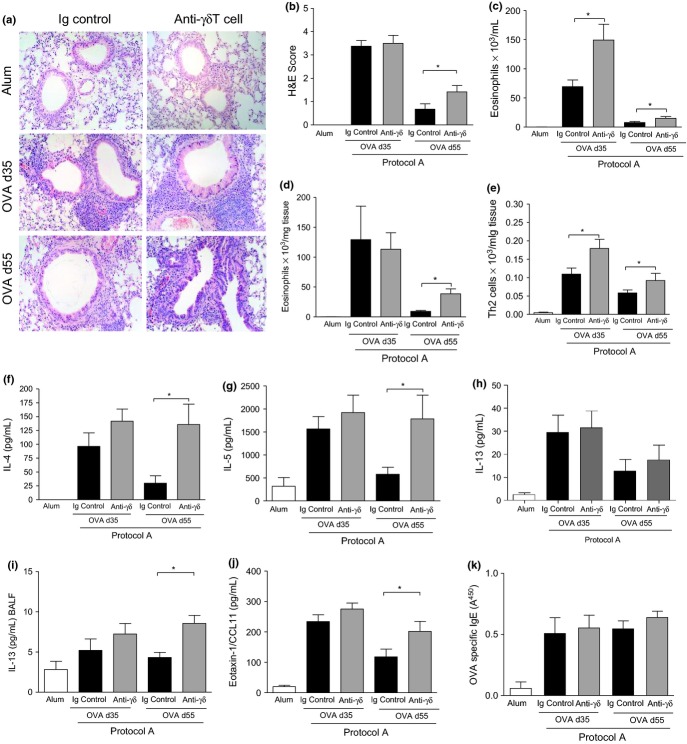
Blockade of γδT cells during established inflammation promotes eosinophilic lung infiltration and elevated pulmonary Th2 responses. Peribronchiolar infiltrates were examined on histological lung sections stained with H&E. Representative photomicrographs at 40× magnification (a). Histopathological scoring of peribronchiolar submucosal lung inflammation (b). Eosinophils were quantified by differential counting of Wright-Giemsa-stained BAL (c) and lung (d) cytospins. Th2 cells, defined as CD3^+^CD4^+^T1/ST2^+^, were evaluated by flow cytometric staining from lung leukocyte preparations (e). Lung IL-4 (f), IL-5 (g) and IL-13 (h), BAL IL-13 (i), lung eotaxin/CCL11 (j) and serum OVA-specific IgE (k) levels assessed by ELISA. Data are mean ± SEM, *n *=* *8–12 mice per group. *P <* *0.05 in comparison with OVA Ig controls (Mann–Whitney *U*-test). Alum controls from days 35 and 55 were not significantly different between time points and are pooled for clarity.

Prolonged OVA challenge is associated with a switch to a chronic inflammatory phenotype which promotes development of remodelling 3. To investigate the participation of γδT cells in this process, we monitored pulmonary eosinophilia following administration of the γδT cell blocking antibody during established disease. Inflammatory infiltrates were evaluated from lung histopathology on days 35 and 55 following OVA challenge (Fig.3a). Immunopathological H&E scoring revealed a degree of inflammatory resolve in OVA control mice between days 35 and 55, in accordance with published reports 3. However, anti-γδTCR-treated mice did not exhibit a similar reduction (Fig.3b). Quantification of cellular infiltrates in the lung compartments demonstrated that anti-γδT cell treatment resulted in exacerbated BAL (Fig.3c) and lung tissue eosinophilia (Fig.3d) on day 55 when compared to sham-treated OVA counterparts.

### γδT cells regulate the CD4^+^Th2 responses during the chronic disease stage

Differentiated Th2 cells, the driving force behind allergic-type pathology in this system, were assessed on day 35 and day 55. Significant numbers of Th2 cells were detected in OVA control mice on day 35; however, the magnitude of this response had reduced by day 55 (Fig.3e). In contrast, the lungs of anti-γδT cell antibody-treated OVA animals had substantially more luminal and lung tissue Th2 lymphocytes at both time points. Th2-associated mediators indicate the magnitude of the ensuing inflammatory response in the airways. Lung Th2 cytokine and chemokine levels were equivalent between groups on day 35. By day 55, mice receiving γδT cell blocking antibody had significantly elevated Th2 cytokines IL-4 and IL-5 (Fig.3f,g). IL-13 levels in the lung were not modulated by neutralisation of γδT cells (Fig.3h), although in the BALF, IL-13 levels were significantly elevated (Fig.3i). Eotaxin-1/CCL11 levels were also augmented compared to Ig control mice in the absence of γδT cells (Fig.3j). In contrast, there was no numerical difference in CD4^+^CD25^+^FoxP3^+^Tregs or the Th1-associated cytokine IFN-γ detected between groups at any time point (data not shown). Despite the increased pulmonary Th2 cytokines in the anti-γδ antibody-treated mice, administration of the blocking antibody had no impact on systemic IgE concentrations (Fig.3k).

### Depletion of γδT cells during the chronic phase affects disease outcome

**Figure 4:**
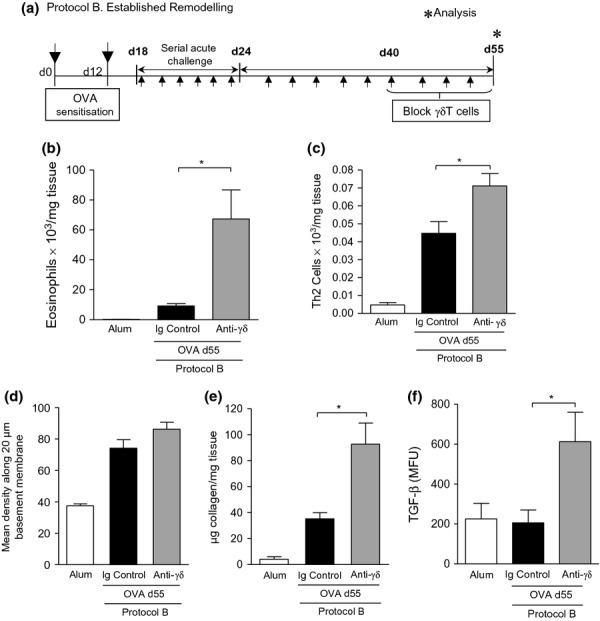
Blocking γδT cells promotes established tissue remodelling in the allergic lung. *Protocol B –* 100 μL of 200 μg/mL anti-TCRδ mAb (or hamster Ig control) was administered i.v. (twice weekly) to female BALB/c mice after establishment of airway remodelling (day 43) and disease parameters assessed on day 55, 24 h after final allergen challenge (a). Lung tissue eosinophils from each group were quantified by differential counting of Wright-Gimsa-stained cytospins (b). Th2 cells, defined as CD3^+^CD4^+^T1/ST2^+^, were evaluated by flow cytometric staining (c). Quantitative analysis of Sirius Red-stained lung sections for subepithelial peribronchiolar collagen density (d). Biochemical Sircol Assay of total lung collagen content (e). Detection of biologically TGF-β activity was measured in lung tissue homogenate using a SEAP reporter bioassay (f). Data are expressed as mean ± SEM. *n *=* *8–12 mice per group. *P <* *0.05 in comparison with OVA controls (Mann–Whitney *U*-test). Alum controls from days 35 and 55 were not significantly different between time points and are pooled for clarity.

Because therapeutic blockade of γδT cells during acute airway inflammation led to exacerbated levels of inflammatory cells and mediators, it is difficult to identify the direct contribution of γδT cells to airway remodelling. To this end, the role of γδT cells during established remodelling was also assessed by delivering anti-TCRδ blocking antibodies from day 40 onwards, when remodelling changes have become established (Protocol B, Fig.4a). Disease parameters were assessed on day 55. Significantly elevated eosinophilia (Fig.4b) and Th2 cells (Fig.4c) were observed in the airways of mice treated with this protocol. No significant difference in subepithelial collagen deposition could be detected (Fig.4d). Total lung collagen content was however significantly amplified in OVA-exposed mice treated with the blocking antibody in comparison with those receiving the control Ig (Fig.4e). The increase in pulmonary collagen was positively correlated with elevated levels of TGF-β in the lung (Fig.4f).

Therapeutic depletion of γδT cells in HDM-induced allergic airway disease.

**Figure 5:**
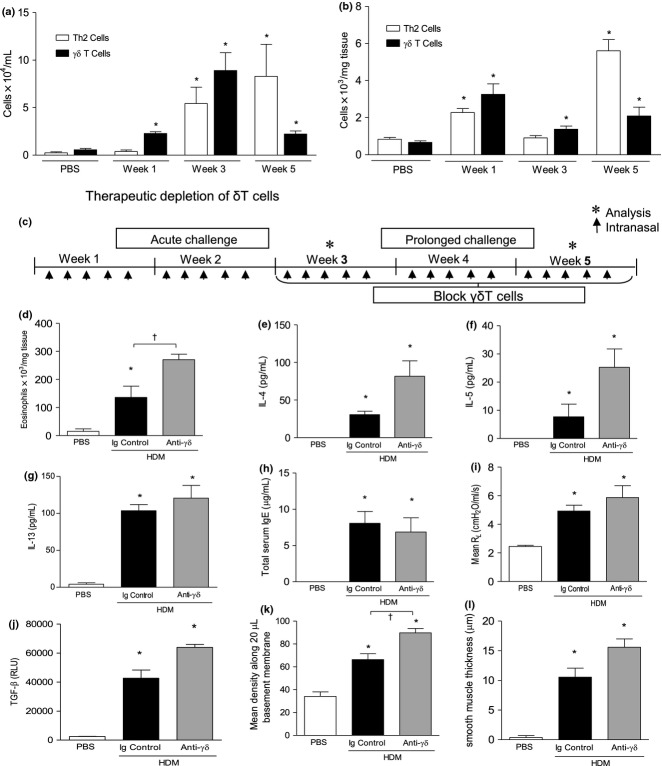
Blockade of γδT cells during HDM-induced allergic airway disease promotes eosinophilic lung infiltration and airway remodelling. TH2 and γδT cells are increased in the BALF (a) and lung (b) following continuous challenge with 25 μg HDM. Following establishment of allergic airway disease, female BALB/c mice were administered 100 μL of 200 μg/mL anti-TCRδ blocking mAb (or hamster Ig control) i.v. (twice weekly) and disease parameters assessed at the end of week 5 (c). Lung tissue eosinophils from each group were quantified by differential counting of Wright-Gimsa-stained cytospins (d). Lung IL-4 (e), IL-5 (f), IL-13 (g) and serum total IgE (h) were measured by ELISA. Airway resistance in response to 30 mg/mL methacholine challenge (i). Detection of biologically active TGF-β activity was measured in lung tissue homogenate using a SEAP reporter bioassay (j). Quantitative analysis of Sirius Red-stained lung sections for subepithelial peribronchiolar collagen density (k). Peribronchiolar smooth muscle thickness (l). Values are expressed as mean ± SEM, *n *=* *8–12 mice per group. *P <* *0.05 in comparison with PBS controls, †*P *<* *0.05 in comparison with HDM Ig controls (Mann–Whitney *U*-test).

The OVA/alum model of allergic airway disease recapitulates many of the clinical features of human asthma and as such is a useful murine model to dissect the cellular pathways involved in disease inception and propagation. However, OVA is not a clinically relevant allergen and the model relies upon peripheral sensitisation in the peritoneal cavity with the adjuvant alum which skews the immune towards a Th2 response. We therefore also investigated the role of pulmonary γδT cells in a model of house-dust mite (HDM)-induced allergic airway disease where sensitisation occurs locally in the lung. Continuous exposure to HDM results in recruitment of Th2 cells and γδT cells (Fig.5a,b). IL-17 levels in the lung were increased fivefold in response to chronic HDM exposure (281 ± 116 vs. 1450 ± 126 pg/mL). The effect of therapeutic blockade of γόT cells on HDM-induced allergic airway (Fig.5c) disease mirrored that previously observed in the OVA model. The number of pulmonary eosinophils was increased in response to allergen challenge with HDM, and eosinophilia was augmented in the absence of functional γόT cells (Fig.5d). IL-4 and IL-5 were also increased in the lung of HDM-exposed mice treated with blocking antibody compared to those injected with the Ig control antibody (Fig.5e,f). However, blockade of γδT cells did not influence pulmonary IL-13 levels in the HDM model at the time point measured (Fig.5g). As observed in the OVA model, anti-γόTCR antibody treatment did not impact immunoglobulin class switching to IgE secretion (Fig.5h). Airway hyperreactivity was also not affected by neutralisation of γδT cells (Fig.5i). Blockade of γδT cells further increased HDM-induced TGF-β levels (Fig.5j) which was associated with an increase in peribronchiolar peribronchial collagen deposition (Fig.5k), as previously observed in the OVA model. Airway smooth muscle mass was elevated in the HDM-exposed mice treated with anti-γόTCR antibody compared to control Ig; however, this was not statistically significant (Fig.5l).

## Discussion

γδT cells are key regulators of pulmonary homeostasis and as such may influence the course and outcome of allergic airway disease 15,18. In the present study, we demonstrate that OVA-sensitised animals and mice continuously exposed to HDM for a prolonged allergen challenge regimen exhibit local expansion of γδT cells in the airways that remains elevated throughout the chronic model. Functional blockade of γδT cells followed by long-term allergen challenge exacerbated airway remodelling with particular reference to ECM deposition. This was accompanied by excessive eosinophilic, Th2 cellular infiltrates and a striking increase in the activity of the profibrotic cytokine, TGF-β in the lungs. Modulation of Tregs and Th1 responses did not play a role in the disease phenotype of anti-TCRδ-treated mice. Prolonged intermittent OVA challenge of sensitised mice results in continual activation of repair responses which are, in part, responsible for the remodelled lung 3,7. Our results show that γδT cells play an important role in regulating established allergic airway inflammation which impacts on development of allergen-driven remodelling.

Chronic asthma is associated with inflammation and structural remodelling of the airways. Infiltrating inflammatory cells such as lymphocytes and eosinophils secrete mediators which promote the development of remodelling 24. IL-5 is a recruitment and survival factor for eosinophils, and blocking IL-5 ameliorates collagen deposition in mice and man indicating a role in airway remodelling 25,26. In particular, eosinophils are a source of TGF-β, an important profibrotic cytokine associated with allergic pathology 27–29. TGF-β directly activates structural cells which drive remodelling, and can induce production of other profibrotic mediators (e.g. PDGF, VEGF). Both eosinophils and TGF-β were elevated following blockade of γδT cells. In the light of this information, impaired resolution of inflammation is likely to be functionally responsible for the exacerbation of airway remodelling.

In the absence of functional γδT cells, enhanced Th2 responses were evident. It has previously been shown that modulation of γδT cells activates αβT cells resulting in marked T cell proliferation. The ligands involved in the cross-talk between γδ- and αβT cells remain undefined, but the data imply that antigen-specific T cells present in the lung following OVA challenge (prior to administration of the functional blocking antibody) proliferate further in the absence of γδT cells which act to constrain T cells of the adaptive immune system and consequently limit production of Th2 cytokines 30. The increase in IL-13 was restricted to the BALF compartment only. Blockade of γδT cells did not augment IL-13 levels in the lung which correlates with lung function and mucous production which were not modulated in the anti-όTCR-treated mice. Vγ4^+^ δT cells are the largest resident population of γδT cells in the adult lung and are induced following allergen challenge. Depletion of this subset of γδT cells has also been associated with an increase in the number of cytokine-producing T cells 31. A potential fibrotic role for the Th2 cytokines IL-4 and IL-13 has been described 29,32,33. IL-13 can induce TGF-β-mediated fibrosis 33, while absence of IL-4 is associated with a reduction of fibrosis in injury models 32.

Regulatory T cells are also involved in regulation of allergic airway inflammation and development of remodelling 34. γδT cells may influence pulmonary regulatory T cell populations. This cannot be excluded as a contributing factor to the observed outcome of chronic-stage disease in the current study. Using a second therapeutic anti-TCRδ administration protocol after remodelling had been initiated (day 43, Protocol B), we also addressed whether γδT cells could influence established airway remodelling. In this situation, γδT cell blockade was also associated with exacerbations of inflammatory and remodelling parameters although these were less severe and the concurrent profibrotic effect less pronounced. This may likely be explained by the shorter duration of γδT cell blockade (12 days vs. 31 days).

Regulation of adaptive immunopathology by γδT cells has been demonstrated in a variety of acute inflammatory disease settings 35. Although several studies have reported a requirement for γδT cells for full development of an acute allergic phenotype 36,37, these have used a prophylactic approach to remove γδT cells. Using a clinically relevant therapeutic approach, the present study demonstrates a regulatory role for these cells during established allergic disease induced experimentally by systemic sensitisation to OVA and also in response to the clinically relevant allergen HDM. The conflicting effects of γδT cells during allergic asthma may be clarified by the functional plasticity and diversity of γδT cells in response to microenvironmental cues 12. During infection, γδT cells display a biphasic stage-dependent response. This corresponds to both promotion of pathogen eradication through potentiating full development of effector responses, followed by a later, immunoregulatory response to restore homeostasis 35. This dual function displays little evidence of antigen specificity and as γδT cells are intimately associated with tissues such as gut, lung and skin where pathogen encounter is greatest and heightened immune defence required is suggestive of a general ‘innate-like’ defence mechanism. Fitting with such a model, we have previously identified an IL-17-secreting subpopulation of γδT cells to play a key role in inflammatory resolution during OVA-induced allergic disease in the lung 18. In the present study, we have shown γδΤ cells remain elevated during chronic HDM challenge concomitant with an increase in pulmonary IL-17 levels (data not shown). γδT cell blockade during prolonged allergen exposure resulted in exacerbated eosinophilia and airway remodelling confirming a regulatory role for these cells in constraining allergen-induced pulmonary pathology. This study clearly illustrates the consequences of impaired resolution on tissue remodelling of the airways following continuous allergen challenge. Overall, the current study strengthens the expanding paradigm of γδT cells as sentinels of epithelial tissues with a key function in minimising persistent inflammation and associated tissue damage observed in so many chronic inflammatory diseases including asthma.

The pulmonary epithelium is the largest mucosal barrier to the environment in the body and, in addition to the underlying mesenchyme, is considered an active player in the allergic response 38,39. γδT cells reside within the epithelium and recognise antigens expressed by stressed/damaged tissue cells. This is thought to facilitate γδT cell-dependent maintenance of tissue integrity 40. In the skin, absence of resident γδ T cells results in a significant delay in wound healing and impaired epidermal cell proliferation 13. Intra-epithelial γδ T cells have also been shown to participate in tissue repair in the gut 41. We have extended these observations and shown that in the lung, mice lacking functional γδT cells exhibit a delay in epithelial regeneration in response to allergen challenge. Mice deficient in γδT cells are also unable to resolve skin wounds, and γδT cells can directly influence bleomycin-induced lung fibrosis 14. Their pro-resolving immunoregulatory function is thought to be aided by a direct effect on wound healing 9 further strengthening the paradigm of γδT cells as effective regulators of tissue repair and epithelial homeostasis 13. Furthermore, γδT cell-induced hyaluronan contributes to resolution of inflammatory responses constituting an active part of immunoregulation to prevent chronic disease 42. Tissue remodelling during chronic inflammatory diseases such as asthma has been considered as a wound healing response. One can speculate that lung remodelling may occur due to ineffective resolution of acute inflammatory episodes triggered by each exposure to allergen. Despite evidence for a direct effect on wound healing, the contribution of γδT cells to regulation of established remodelling in this study cannot be uncoupled from their regulatory influence during established disease as inflammation, a contributor to remodelling, is also exacerbated in the absence of γδT cells.

The data presented in the present study demonstrate for the first time that γδT cells are upregulated during chronic allergic airway disease induced both systemically and locally in the lung and have the profound ability to affect the natural resolution of chronic inflammation and influence the development of remodelling. Furthermore, γδT cells are able to affect responses even when allergic disease is established. An important role for γδT cells in regulating inflammation has previously been implied during acute infectious inflammation. The information presented in the present study strengthens the expanding paradigm of γδT cells as regulators of inflammation-associated tissue injury. The role of γδT cells in asthma pathogenesis remains unclear. During symptomatic asthma exacerbations, γδT cells are reportedly increased 43–45. However, there are also patient data indicating that there is no change in γδT cells in asthmatic cohorts compared with non-asthmatics 46,47 and that γδT cells are reduced in allergic asthma compared with healthy controls 48. It is tempting to speculate based on the results of the present study that in some subsets of patients with asthma, a failure to upregulate γδT cells may result in an uncontrolled eosinophilic and Th2-mediated immune response with dysregulated ECM deposition and airway remodelling. The increased understanding of factors regulating chronic inflammation and its contribution to the pathophysiological features of allergic disease may open up new avenues for future anti-inflammatory asthma treatment strategies.

## References

[b1] Bousquet J (2000). Relating inflammatory changes in asthma to clinical status. Respir Med.

[b2] Robinson DS, Hamid Q, Ying S (1992). Predominant TH2-like bronchoalveolar T-lymphocyte population in atopic asthma. N Engl J Med.

[b3] McMillan SJ, Lloyd CM (2004). Prolonged allergen challenge in mice leads to persistent airway remodelling. Clin Exp Allergy.

[b4] Green RH, Brightling CE, McKenna S (2002). Asthma exacerbations and sputum eosinophil counts: a randomised controlled trial. Lancet.

[b5] Jatakanon A, Lim S, Barnes PJ (2000). Changes in sputum eosinophils predict loss of asthma control. Am J Respir Crit Care Med.

[b6] Vignola AM, Chanez P, Siena L, Chiappara G, Bonsignore G, Bousquet J (1998). Airways remodelling in asthma. Pulm Pharmacol Ther.

[b7] Chiappara G, Gagliardo R, Siena A (2001). Airway remodelling in the pathogenesis of asthma. Curr Opin Allergy Clin Immunol.

[b8] Ward C, Walters H (2005). Airway wall remodelling: the influence of corticosteroids. Curr Opin Allergy Clin Immunol.

[b9] Jameson J, Havran WL (2007). Skin gammadelta T-cell functions in homeostasis and wound healing. Immunol Rev.

[b10] Born WK, Lahn M, Takeda K, Kanehiro A, O'brien RL, Gelfand EW (2000). Role of gammadelta T cells in protecting normal airway function. Respir Res.

[b11] Chien YH, Konigshofer Y (2007). Antigen recognition by gammadelta T cells. Immunol Rev.

[b12] Strid J, Sobolev O, Zafirova B, Polic B, Hayday A (2011). The intraepithelial T cell response to NKG2D-ligands links lymphoid stress surveillance to atopy. Science.

[b13] Jameson J, Ugarte K, Chen N (2002). A role for skin gammadelta T cells in wound repair. Science.

[b14] Braun RK, Ferrick C, Neubauer P (2008). IL-17 producing gammadelta T cells are required for a controlled inflammatory response after bleomycin-induced lung injury. Inflammation.

[b15] Lahn M, Kanehiro A, Takeda K (2001). gammadelta T cells as regulators of airway hyperresponsiveness. Int Arch Allergy Immunol.

[b16] McMenamin C, Pimm C, McKersey M, Holt PG (1994). Regulation of IgE responses to inhaled antigen in mice by antigen-specific gamma delta T cells. Science.

[b17] McMenamin C, McKersey M, Kuhnlein P, Hunig T, Holt PG (1995). Gamma delta T cells down-regulate primary IgE responses in rats to inhaled soluble protein antigens. J Immunol.

[b18] Murdoch JR, Lloyd CM (2010). Resolution of allergic airway inflammation and airway hyperreactivity is mediated by IL-17-producing {gamma}{delta}T cells. Am J Respir Crit Care Med.

[b19] Martin TR, Gerard NP, Galli SJ, Drazen JM (1988). Pulmonary responses to bronchoconstrictor agonists in the mouse. J Appl Physiol.

[b20] Hamelmann E, Schwarze J, Takeda K (1997). Noninvasive measurement of airway responsiveness in allergic mice using barometric plethysmography. Am J Respir Crit Care Med.

[b21] Humbles AA, Lloyd CM, McMillan SJ (2004). A critical role for eosinophils in allergic airways remodeling. Science.

[b22] Tesseur I, Zou K, Berber E, Zhang H, Wyss-Coray T (2006). Highly sensitive and specific bioassay for measuring bioactive TGF-beta. BMC Cell Biol.

[b23] Lahn M, Kanehiro A, Takeda K (2002). MHC class I-dependent Vgamma4 + pulmonary T cells regulate alpha beta T cell-independent airway responsiveness. PNAS.

[b24] Trivedi SG, Lloyd CM (2007). Eosinophils in the pathogenesis of allergic airways disease. Cell Mol Life Sci.

[b25] Cho JY, Miller M, Baek KJ (2004). Inhibition of airway remodeling in IL-5-deficient mice. J Clin Invest.

[b26] Flood-Page P, Menzies-Gow A, Phipps S (2003). Anti-IL-5 treatment reduces deposition of ECM proteins in the bronchial subepithelial basement membrane of mild atopic asthmatics. J Clin Invest.

[b27] McMillan SJ, Xanthou G, Lloyd CM (2005). Manipulation of allergen-induced airway remodeling by treatment with anti-TGF-beta antibody: effect on the Smad signaling pathway. J Immunol.

[b28] Bonniaud P, Margetts PJ, Ask K, Flanders K, Gauldie J, Kolb M (2005). TGF-beta and Smad3 signaling link inflammation to chronic fibrogenesis. J Immunol.

[b29] Richter A, Puddicombe SM, Lordan JL (2001). The contribution of interleukin (IL)-4 and IL-13 to the epithelial-mesenchymal trophic unit in asthma. Am J Respir Cell Mol Biol.

[b30] Kaufmann SH, Blum C, Yamamoto S (1993). Crosstalk between alpha/beta T cells and gamma/delta T cells in vivo: activation of alpha/beta T cell responses after gamma/delta T-cell modulation with the monoclonal antibody GL3. PNAS.

[b31] Hahn YS, Taube C, Jin N (2003). V gamma delta T cells regulate airway hyperreactivity to methacholine in ovalbumin-sensitized and challenged mice. J Immunol.

[b32] Huaux F, Liu T, McGarry B, Ullenbruch M, Phan SH (2003). Dual roles of IL-4 in lung injury and fibrosis. J Immunol.

[b33] Lee CG, Homer RJ, Zhu Z (2001). Interleukin-13 induces tissue fibrosis by selectively stimulating and activating transforming growth factor beta(1). J Exp Med.

[b34] Kearley J, Robinson DS, Lloyd CM (2008). CD4+ CD25+ regulatory T cells reverse established allergic airway inflammation and prevent airway remodeling. J Allergy Clin Immunol.

[b35] Carding SR, Egan PJ (2002). Gammadelta T cells: functional plasticity and heterogeneity. Nat Rev Immunol.

[b36] Schramm CM, Puddington L, Yiamouyiannis CA (2000). Proinflammatory roles of T-cell receptor (TCR)gammadelta and TCRalphabeta lymphocytes in a murine model of asthma. Am J Respir Cell Mol Biol.

[b37] Zuany-Amorim C, Ruffie C, Haile S, Vargaftig BB, Pereira P, Pretolani M (1998). Requirement for gammadelta T cells in allergic airway inflammation. Science.

[b38] Holgate ST, Davies DE, Puddicombe S (2003). Mechanisms of airway epithelial damage: epithelial-mesenchymal interactions in the pathogenesis of asthma. Eur Respir J Suppl.

[b39] Hammad H, Lambrecht BN (2008). Dendritic cells and epithelial cells: linking innate and adaptive immunity in asthma. Nat Rev Immunol.

[b40] Groh V, Steinle A, Bauer S, Spies T (1998). Recognition of stress-induced MHC molecules by intestinal epithelial gammadelta T cells. Science.

[b41] Chen Y, Chou K, Fuchs E, Havran WL, Boismenu R (2002). Protection of the intestinal mucosa by intraepithelial gamma delta T cells. PNAS.

[b42] Jameson JM, Cauvi G, Sharp LL, Witherden DA, Havran WL (2005). Gammadelta T cell-induced hyaluronan production by epithelial cells regulates inflammation. J Exp Med.

[b43] Spinozzi F, Agea E, Bistoni O (1996). Increased allergen-specific, steroid-sensitive gamma delta T cells in bronchoalveolar lavage fluid from patients with asthma. Ann Intern Med.

[b44] Pawankar R (2000). Gammadelta T cells in allergic airway diseases. Clin Exp Allergy.

[b45] Molfino NA, Doherty PJ, Suurmann IL (1996). Analysis of the T cell receptor Vgamma region gene repertoire in bronchoalveolar lavage (BAL) and peripheral blood of atopic asthmatics and healthy subjects. Clin Exp Immunol.

[b46] Walker C, Bode E, Boer L, Hansel TT, Blaser K, Virchow JC (1992). Allergic and nonallergic asthmatics have distinct patterns of T-cell activation and cytokine production in peripheral blood and bronchoalveolar lavage. Am Rev Respir Dis.

[b47] Fajac I, Roisman GL, Lacronique J, Polla BS, Dusser DJ (1997). Bronchial gamma delta T-lymphocytes and expression of heat shock proteins in mild asthma. Eur Respir J.

[b48] Zhao Y, Yang J, Gao YD (2011). Altered expressions of helper T cell (Th)1, Th2, and Th17 cytokines in CD8(+) and γό T cells in patients with allergic asthma. J Asthma.

